# Utility of diagnostic tests in vomiting dogs presented to an internal medicine emergency service

**DOI:** 10.3389/fvets.2023.1063080

**Published:** 2023-02-02

**Authors:** Bettina Holzmann, Melanie Werner, Stefan Unterer, René Dörfelt

**Affiliations:** ^1^Clinic of Small Animal Medicine, Centre for Clinical Veterinary Medicine, Ludwig-Maximilians University, Munich, Germany; ^2^Clinic for Small Animal Internal Medicine, Vetsuisse Faculty, University of Zurich, Zurich, Switzerland

**Keywords:** ultrasound, radiography, blood gas analysis, self-limiting vomiting, canine

## Abstract

**Introduction:**

Vomiting is a common sign in dogs presenting to emergency services. It can be self-limiting, a sign of a life-threatening extraintestinal, or intestinal disorder. Reasonable diagnostics should be performed to determine the underlying cause. This study aimed to assess the utility of diagnostic tests in vomiting dogs, and its correlation with patient history, and physical examination results. Additionally, parameters to differentiate uncomplicated vomiting from complicated vomiting were investigated.

**Methods:**

In this prospective, observational, clinical study, data from 99 client-owned dogs with vomiting, presenting as first opinion cases, were evaluated. History, physical examination, duration of clinical signs, overall number of episodes of vomiting, appetite, and additional clinical signs were recorded. The standardized diagnostic evaluation of all patients included venous blood gas analysis, complete blood count, serum biochemistry profile, canine pancreatic lipase, abdominal radiographs, ultrasound, and urinalysis. Follow-up was performed 4–5 days later. Based on severity of disease and clinical course, dogs were categorized to “uncomplicated vomiting” (UN), or “complicated vomiting” (COM). The utility of each test for diagnosing the cause of vomiting was evaluated. Spearman correlation coefficient, Chi-squared-, unpaired t-, and Mann–Whitney U-test were used. Statistical significance was defined as p ≤ 0.05.

**Results:**

Out of the 99 dogs, 34 had uncomplicated courses of disease (UN). In 60/99 cases, a diagnosis was obtained, and in 39/99 cases, the cause for vomiting remained unknown. Longer duration of clinical signs, and reduced appetite were associated with higher utility of abdominal ultrasound. A poor mentation was associated with a higher utility of blood examinations and abdominal radiographs. Dogs presenting with an impaired mentation or with additional clinical signs other than diarrhea, were more likely to be in the COM group.

**Discussion:**

Based on this investigation, general recommendations concerning the diagnostic approach for patients with vomiting could not be provided. For dogs who have exclusively vomiting as a clinical sign, and present in good mentation, further investigations might not be beneficial, and these dogs may recover with symptomatic treatment alone. Additional diagnostics could be indicated in dogs with additional clinical signs other than diarrhea.

## 1. Introduction

Vomiting is a common clinical sign in dogs, presented to emergency services. It may be self-limiting or may progress to chronic disorders or even life-threatening conditions. Approximately 19% of dogs in a normal dog population showed vomiting at least once during a two-week period (1).

Multiple differential diagnoses must be considered in vomiting dogs. In a study involving 213 dogs, the following conditions were reported as the most common causes of vomiting: gastrointestinal disorders, such as gastroenteritis or gastrointestinal foreign bodies, followed by systemic metabolic or toxic disorders, such as uremia, diabetic ketoacidosis, electrolyte disturbances, or intoxication (including drugs). Besides, non-gastrointestinal abdominal disorders such as pancreatic and hepatobiliary disorders or peritonitis, followed by neurological problems were documented as etiologies (2).

When vomiting is caused by an underlying severe local or systemic disorders patients require intensive treatment or surgical intervention. Identifying these specific cases at presentation can be challenging. The main goal of a rational workup of vomiting patients is to rapidly exclude or detect life threatening conditions or surgical indications as well as to select valuable and reasonable diagnostics. This is often based on empirical decisions, and inexperienced veterinarians can be uncertain about the correct diagnostic approach. It is in the owner's and veterinarian's best interest to perform adequate tests for an individual patient. However, the tests should be time- and economically efficient.

A recently published study demonstrated that a higher age and a higher frequency of vomiting was correlated with a better utility of sonography in dogs with chronic vomiting (3). However, another study reported, that radiography and ultrasound did not assist diagnosis in vomiting dogs in **~**75 and 45% respectively (2).

Till date, none of the studies have investigated and compared the diagnostic utility of different tests in vomiting dogs with a standardized diagnostic approach. Furthermore, there is no data regarding the discrimination between cases of uncomplicated and complicated vomiting on the basis of parameters of the patient history and clinical presentation, or diagnostic values.

This prospective study aimed to describe the most prevalent causes of vomiting in dogs presented in an internal medicine emergency setting, and to assess the utility of different diagnostic tests in dogs with vomiting. In addition, the potential influence of anamnestic and clinical parameters on the utility of diagnostic tests should be determined. A second goal was to find parameters helping to differentiate uncomplicated from complicated cases of vomiting.

We hypothesized that the diagnostic utility of the performed tests would increase in patients with severe clinical signs. The presence of dehydration, severe abdominal pain, or additional clinical signs at presentation was expected to be predictive of complicated vomiting. Evidence of systemic disorders, for example polyuria and polydipsia (PU/PD), or abnormal rectal temperature was suspected to be associated with a higher diagnostic utility of laboratory tests. A longer duration of vomiting, was expected to be concomitant with a higher utility of imaging.

## 2. Material and methods

The study protocol was approved by the ethics committee of the Center of Clinical Veterinary Medicine (111-04-02-2018). Informed consent was obtained from all owners. Over a period of two years, client-owned dogs presenting with vomiting at an internal medicine emergency service, were enrolled in this prospective, observational, clinical study. Exclusively first-opinion cases were enrolled in the study. Patients were excluded if they had already been treated for their presenting condition prior to presentation, or if follow-up data were not available.

Detailed anamnestic information was gathered, with special attention to the time between the onset of vomiting and presentation, overall number of vomiting episodes, appetite (0 = normal, 1 = mild reduced, 2 = moderately reduced, 3 = severely reduced), and additional clinical signs. A complete physical examination was performed, and the results were documented for all dogs at the time of presentation. The following parameters were selected for evaluation: mentation (0 = normal, 1 = mildly reduced, 2 = moderately reduced, 3 = severely reduced), rectal temperature (categorized as hypo-, normo-, or hyperthermic; < 38.0°C, 38.0–39.0 °C, >39.0 °C), percental dehydration status (evaluated on the basis of mucous membrane moisture, skin turgor, and position of bulbi), and abdominal tension (0 = soft, 1 = mildly tense, 2 = moderately tense, 3 = severely tense). The attending clinician estimated overall pain using a visual analog scale with a score from 1 (lowest) to 10 (highest level of pain). Patients were considered in shock if at least two of the following parameters were detected: increased heart rate (> 120 bpm), red or pale mucous membranes, shortened ( ≤ 1 s) or prolonged (>2 s) capillary refill time, decreased pulse quality, and cold extremity temperature.

Standardized diagnostic evaluation for all patients included imaging and laboratory tests. Radiographs of the abdomen in right laterolateral and ventrodorsal views were recorded for every dog (FDR Smart X, Fuji Film, Minato, Japan) and assessed by a non-board-certified emergency clinician. This clinician also performed a standardized abdominal ultrasound examination to evaluate intra-abdominal organ echogenicity, size and shape, thickness, layering of gastric and intestinal walls, filling of the gastrointestinal tract, and the presence or absence of abdominal effusion (LOGIQ P6, GE Healthcare, Chicago, USA). Urinalysis (SediVue Dx, IDEXX Laboratories, Westbrook, USA^)^ was performed, including a dip stick and evaluation of urine sediment. Venous blood gas analysis (Rapidpoint 405, Siemens Healthcare GmbH, Erlangen, Germany), complete blood count (XT 2000i, Sysmex GmbH, Kobe, Japan), serum biochemistry profile (Cobas Integra 400 plus analyzer, Roche diagnostics, Rotkreuz, Switzerland), and canine pancreas-specific lipase rapid test (SNAP cPl, IDEXX Laboratories, Westbrook, USA). A normal SNAP cPL result was rated as an exclusion of pancreatitis. In case of an abnormal SNAP cPL result, samples were subjected to quantitative assessment of specific canine pancreatic lipase (spec cPL, IDEXX Europa B.V., Hoofddrorp, Netherlands). Spec cPL values over 400 μg/L in combination with ultrasonographic abnormalities of the pancreatic region confirmed pancreatitis.

A diagnosis of acute hemorrhagic diarrhea syndrome (AHDS) was made in dogs with acute onset of hemorrhagic diarrhea. Drugs or toxins causing mucosal irritation (e.g., doxycycline, NSAIDs), previous events causing intestinal damage (e.g., blood loss, heat stroke), acute liver or kidney failure, acute pancreatitis, gastrointestinal obstruction, giardia-infection or other gastrointestinal parasites had to be excluded in these patients.

The utility of each diagnostic test for diagnosis of the cause of vomiting was evaluated using the following scale adapted from a previous study (3):

1. The test obtained the diagnosis.
2. The test provided very important information or helped reach a diagnosis. This was crucial for diagnosis.
3. The test provided information that helped assess other data or make a diagnosis.
4. The test provided descriptive information that did not affect the diagnosis. The same diagnosis would have been made without performing the test.
5. The test provided conflicting information, that did not support or hindered the diagnosis.

In case of out-patient treatment, the owner was contacted for follow-up *via* phone or email 4–5 days after the initial presentation. Dogs were assigned to the group “uncomplicated vomiting” (UN) if only symptomatic treatment as defined below led to recovery within this time, and if dogs remained asymptomatic until the time of follow-up even after discontinuing antiemetic therapy.

Dogs were classified to the group “complicated vomiting” (COM) when further investigation, surgery, or more intensive support, that exceeded symptomatic treatment as defined below, were required. If a dog had to be hospitalized, presented for further treatment or had not recovered until follow-up, it was classified as complicated vomiting.

Medication was not standardized for the dogs included in the study. While all patients were received maropitant, additional medications, such as analgesics, antibiotics, and gastrointestinal protectants, were selected individually, depending on the diagnostic results. Symptomatic treatment included administration of one antiemetic drug for a maximum of 2 days, and if considered appropriate by the attending clinician, short-time intravenous or subcutaneous infusion-therapy, analgesics, gastrointestinal protectants, and probiotics.

### 2.1. Data analysis and statistics

Statistical analysis was performed with a commercial program (SPSS Statistics version 26, IBM, Armonk, USA).

Normality of the data was analyzed using the Kolmogorov-Smirnov test. Normally distributed data are presented as mean (m) ± standard deviation (SD) and non-normally distributed data were presented as medians and ranges. To investigate the correlation between the diagnostic utility of different tests (blood examination, abdominal radiographs, ultrasonography, urinalysis, SNAP cPL) and ordinal or metric parameters like age, duration of clinical signs, overall number of vomiting episodes, mentation, abdominal tension, temperature, dehydration, and pain, the Spearman correlation coefficient was calculated. Furthermore, for nominal parameters (additional clinical signs), the Chi-squared test was performed.

To evaluate the differences in parameters between the COM and UN groups for normally distributed data, an unpaired *t*-test was performed. For non-normally distributed data, the Mann-Whitney U-test was used. For ordinal parameters, the Chi-squared test was performed to detect differences between the two groups. Statistical significance was defined as *P* ≤ 0.05. Due to the explorative nature of this study, *p*-values were not adjusted for multiple comparisons to reduce the probability of Type II error.

## 3. Results

A total of 101 dogs were included in this study. Two dogs were lost to follow-up and excluded from the analysis. Out of the 99 dogs completing the study, 54 (55%) were female (31 spayed and 23 intact), and 45 (45%) were male (15 neutered and 30 intact). The most common breed was mixed breed (34/99; 34%), followed by Dachshund (7/99; 7%), Labrador Retriever (4/99; 4%), Maltese (4/99; 4%), Jack Russel Terrier (3/99; 3%), Chihuahua (3/99; 3%), and Bichon Frisé (3/99; 3%). Other breeds had less than three individuals.

The median age was 5.0 years (range 0.2–16.0 years), with 6/99 dogs being under 6 months of age and 13/99 dogs being 10 years or older. The bigger part of the study population (59/99; 60%) were young and middle-aged adult dogs (age 1–10 years). The median body weight was 9.7 kg (range 1.9–43.0 kg). Prior to presentation, the median duration of vomiting was 8 hours (range, 1–330 h), and the median number of vomiting episodes was 5 (range, 1–28).

The most common additional clinical sign was diarrhea (61/99; 61%), which was hemorrhagic in 31/61 (51%) dogs. A total of five dogs (5%) exhibited polyuria/polydipsia, and two of these had diarrhea. Tremor and ataxia, dyspnea, and pruritus were displayed each by one dog. Thirty-two of 99 (32%) dogs presented with vomiting as the only clinical sign.

In 34/99 (34%) dogs, vomiting resolved with symptomatic treatment within 4–5 days (group UN), while 65/99 (66%) dogs were categorized into the group COM. A definite diagnosis was made in 60/99 (61%) dogs (Table 1). In 30/34 (88%) cases in group UN and 9/65 (14%) cases in group COM, no underlying disorder could be identified with the performed diagnostics. These cases were defined as uncomplicated or complicated vomiting of unknown origin.

**Table 1:** Diagnosis of 34/99 dogs with uncomplicated vomiting and 65/99 dogs with complicated vomiting.

**Diagnosis**	**Uncomplicated vomiting (*n*)**	**Complicated vomiting (*n*)**
Total	34	65
Anaphylaxis		1
Adhesive ileus		1
Acute hemorrhagic diarrhea syndrome		15
Pyelonephritis		2
Foreign body ileus		3
NSAID-associated gastrointestinal hemorrhage		3
Giardiasis		3
Heat stroke		1
Hepatopathy		1
Ileum coprostasis		2
Intestinal lymph node abscess		1
Intestinal lymphoma		1
Pyometra		2
Non-obstructive foreign body	1	3
Pancreatitis	3	13
Pesticide toxicity		1
Protein-losing nephropathy		1
Undefined neoplasia		1
Undefined Intoxication		1
Unknown cause	30	9

In 24/60 (40%) cases, the diagnosis was obtained by one of the standardized diagnostic tests. In 8/60 (13%) cases with a definite diagnosis, other diagnostic examinations, such as cytology, (exploratory) laparotomy, bacterial culture or fecal examination were required. In 4/60 (6%) cases, diagnosis was made based on clinical presentation and patient history (heat stroke, anaphylaxis, intoxication). Acute hemorrhagic diarrhea syndrome (AHDS) was diagnosed in 15/60 cases (25%) according to the previously defined clinical criteria. In the 9/65 (14%) remaining cases, a combination of different tests and clinical data led to a definite diagnosis.

One dog with clinical assessment of uncomplicated vomiting was hospitalized for one night at the owner's request. All other dogs in this group (33/34; 97%) were treated as outpatients with symptomatic treatment. In group COM, 53/65 (81.5%) dogs were hospitalized, while 12/65 (18%) were treated as outpatients, and were re-presented to the clinic or to the referring veterinarian for further diagnostic workup or treatment. A total of seven dogs from group COM (7/65; 11%) were referred for surgery to address the underlying problem. Two of these dogs (2/7; 29%) died due to intra- and postoperative complications (intraoperative cardiopulmonary arrest in one dog with foreign body ileus and suspected postoperative intra-abdominal steatitis in one dog with ileal coprostasis). Two dogs (2/65; 3%) were euthanized because of poor prognosis of the underlying disorders (intestinal lymphoma, end stage liver disease).

### 3.1. Relationship between diagnostic parameters and utility score

#### 3.1.1. Primary blood examination

Blood examinations enabled diagnosis in one dog [1/99; 1%; non-steroidal anti-inflammatory drug (NSAID)-associated gastrointestinal hemorrhage] and did not affect diagnosis in 70/99 (71%) dogs (Table 2).

**Table 2:** Utility score of different diagnostic tests in 99 dogs with vomiting.

**Utility score**	**Blood examination**	**Radiography**	**Urinalysis**	**Sonography**	**SNAP cPL**
	***N***	***N***	***N***	***N***	***N***
1	1	3	1	3	16
2	3	3	4	14	0
3	23	2	0	19	2
4	70	87	82	57	70
5	2	4	12	6	11
Median	4	4	4	4	4

Utility of blood examination had a weak negative correlation with mentation (*P* = 0.011, r = −0.255). This finding implicates superior utility of blood examinations in dogs with impaired mentation.

There was a significant connection between the utility of blood test and attending clinical signs (*P* = 0.028). There was a greater utility of blood test in dogs that showed additional clinical signs other than non-hemorrhagic diarrhea.

#### 3.1.2. Abdominal radiographs

Radiographs confirmed the diagnosis in 3/99 (3%) dogs (2 foreign body ileus, 1 ileal coprostasis). In 87/99 (88%) dogs, the same diagnosis could have been made without radiographs (Table 2). In one case of an obstructive foreign body, radiographs were assessed as non-obstructive, and were therefore misleading.

There was a significant negative correlation between the utility of radiographs and the patients' mentation (P = 0.027; r = −0.222; Table 3).

**Table 3:** Spearman correlation between clinical parameters and utility score of different diagnostic tests in 99 dogs with vomiting.

**Parameter**	**Blood examination**		**Radiography**		**Urinalysis**		**Sonography**		**SNAP cPL**	
	***r***	***p***	***r***	***p***	***r***	***p***	***r***	***p***	***r***	***p***
Age	−0.143	0.158	−0.013	0.764	−0.048	0.636	−0.207	0.291	−0.115	0.257
Duration	−0.04	0.691	−0.086	0.397	−0.014	0.891	**−0.237**	**0.018**	0.044	0.666
Frequency	0.128	0.208	0.146	0.148	−0.045	0.656	0.042	0.678	−0.101	0.321
Appetite	0.055	0.587	0.032	0.751	−0.080	0.431	**−0.257**	**0.010**	−0.018	0.860
Mentation	**−0.255**	**0.011**	**−0.222**	**0.027**	−0.126	0.213	−0.054	0.598	0.053	0.601
Abdominal tension	−0.022	0.823	0.039	0.700	−0.080	0.433	0.143	0.157	0.054	0.596
Temperature	0.086	0.398	0.002	0.982	−0.015	0.880	−0.104	0.304	−0.120	0.237
Dehydration	−0.166	0.101	−0.045	0.661	0.135	0.181	−0.078	0.441	−0.042	0.680
Pain	−0.111	0.272	0.004	0.966	−0.030	0.771	0.116	0.251	0.086	0.398

The bold values are statistically significant (*p* < 0.05).

p, significance; r, correlation coefficient; SNAP cPL, SNAP canine pancreatic lipase.

#### 3.1.3. Urinalysis

Diagnosis was obtained *via* urinalysis in one dog (pyelonephritis), and provided important information in 4/99 (4%) patients. In most cases (82/99, 83%), urinalysis did not help in establishing a diagnosis, and in 12/99 (12%) cases, the results of urinalysis were misleading. Proteinuria (7/12, 58%), ketones (5/12, 42%), and glucosuria (1/12, 8%) were detected *via* urine sticks, and bacteriuria was reported in 8/12 (67%) dogs. In 10/12 (83%) dogs, a combination of these findings led to misinterpretation, as there was no underlying renal or urinary disorder explaining vomiting in any of these cases.

No significant correlation was observed between the diagnostic utility of urinalysis and any of the parameters (Table 3).

#### 3.1.4. Ultrasound

Abdominal ultrasonography obtained the diagnosis in one case of an obstructive intestinal foreign body and in two cases of pyometra. In one case of foreign body ileus, sonography was crucial for diagnosis, as the diagnosis was made based on a combination of sonographic and radiographic findings. In the third case of foreign body ileus, sonography was misleading as no signs of obstruction were observed.

Abdominal ultrasound was crucial or helpful to make a diagnosis (Score 2 and 3) in 33/99 (33%) dogs.

The diagnostic utility of ultrasound was weakly negatively correlated with the patient's appetite (*P* = 0.010, r = −0.257) and the duration of vomiting (*P* = 0.018, r = −0.237). A negative correlation indicates that the diagnostic utility of abdominal ultrasound increases with a longer duration of vomiting and more impaired appetite (Table 3).

#### 3.1.5. SNAP cPL

SNAP cPL obtained the diagnosis of pancreatitis in 16/99 (16%) cases, supported by elevated spec cPL levels and sonographic findings. SNAP cPL was positive in 29/99 cases. In 7/29 cases (24%), spec cPL levels were low and in one dog with spec cPL over 400 μg/l no sonographic evidence of pancreatitis was detected. SNAP cPL was rated as misleading in these cases. In two dogs with high spec cPL levels, pancreatic enzyme leakage was believed to be secondary (giardiasis and hepatopathy).

No significant correlations were detected with any of the parameters investigated (Table 3).

### 3.2. Differentiation between uncomplicated and complicated vomiting

#### 3.2.1. History

**Figure 1:**
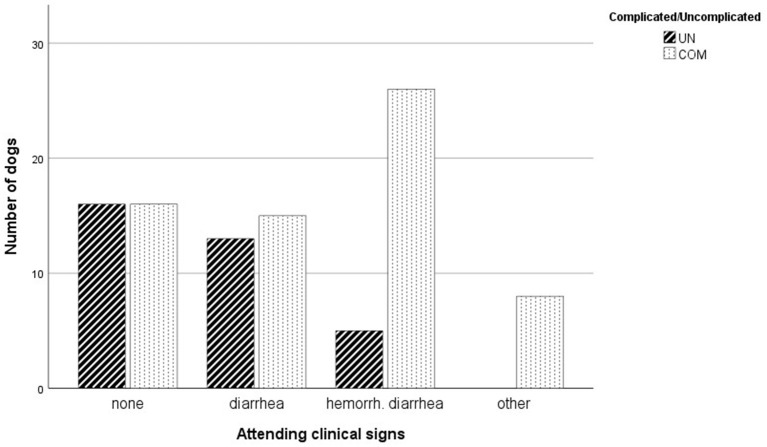
Attending clinical signs in dogs with uncomplicated (UN) vomiting compared to complicated vomiting (COM). Abbreviation: hemorrh. diarrhea, hemorrhagic diarrhea.

There were significantly more dogs with additional clinical signs in the COM group than in the UN group (*P* = 0.023; Table 4). If dogs had additional clinical signs other than non-hemorrhagic diarrhea, they were likely to have complicated vomiting (*p* < 0.001). Dogs presenting with dyspnea, PU/PD, or ataxia were all assigned to COM and only 5/31 (16%) dogs with hemorrhagic diarrhea had uncomplicated vomiting (Figure 1).

**Table 4:** Comparison of demographic, anamnestic, and clinical parameters of 34 dogs with uncomplicated vomiting and 65 dogs with complicated vomiting.

**Parameter**	**Uncomplicated**	**Complicated**	***p*-value**
	**(*****n*** = **34)**	**(*****n*** = **65)**	
Age (years)	4 (0.2 – 13.5)	5.5 (0.2–16)	0.632
Duration (h)	8 (1–90)	8 (1–330)	0.822
Appetite (0/1/2/3)	11/4/8/11	16/9/20/20	0.802
Vomiting episodes	4.5 (1–15)	5 (1–28)	0.301
Additional clinical signs	18 (53%)	50 (76%)	**0.023**
Mentation (0/1/2/3)	7/26/1/0	11/31/19/4	**0.004**
Temperature (hypo-/normo-/hyperthermic)	1/29/4	12/69/18	**0.037**
Abdominal tension (0/1/2/3)	7/17/9/1	16/12/27/9	**0.013**
Heart rate (/min)	110 (56–200)	120 (68–220)	**0.015**
Dehydration (%)	6 (0–8)	6 (0–9)	**0.022**
Pain score	4 (0–7)	5 (1–9)	**0.019**

Values are presented as median and range or absolute number of dogs. *P*-values in bold are significant.

#### 3.2.2. Physical examination

There was a significant difference in the mentation of patients between the two groups (*p* = 0.004). Dogs with a moderately or severely reduced mentation (*P* = 0.004) were more likely to have complicated vomiting. Dogs with complicated vomiting had significantly increased abdominal tension (*P* = 0.013), increased pain score (*P* = 0.019), and a higher heart rate (*P* = 0.015) than those with uncomplicated vomiting. Additionally, dogs with complicated vomiting were significantly more dehydrated (*P* = 0.022) than those with uncomplicated vomiting. There were significantly more dogs with abnormal body-temperature in group COM than in group UN (*P* = 0.037; Table 4). A total of 30/99 dogs (30%) were either hypo- or hyperthermic (12/30; 40% and 18/30; 60%). Out of these dogs, only 5/30 (17%) were assigned to group UN. A total of 26/99 dogs (26%) were presented with shock and all of these dogs were classified as complicated vomiting.

#### 3.2.3. Initial blood examination

Only Alkaline phosphatase was significantly higher in the COM (*p* = 0.024, Table 5) than in the UN group.

**Table 5:** Comparison of the results of venous blood gas and hematologic and biochemistry profile of 34 dogs with uncomplicated vomiting and 65 dogs with complicated vomiting.

**Parameter**	**Uncomplicated**	**Complicated**	***p*-value**
pH- Status (azidemia/normal/alkalemia)	2/32/9	6/59/9	0.562
Chloride (mmol/l)	109 (103–114)	110 (99–118)	0.719
Potassium (mmol/l)	3.87 ± 0.27	3.99 ± 0.39	0.103
Sodium (mmol/l)	145 ± 2.7	145 ± 3.8	0.817
Lactate (mmol/l)	1.9 (0.8–7.5)	2.2 (0.8–6.0)	0.155
Hematocrit (l/l)	0.52 ± 0.07	0.53 ± 0.09	0.517
Leucocyte counts (G/l)	12.1 (5.86–27.4)	9.8 (6.5–25.5)	0.517
Neutrophil count (G/l)	9.56 (3.63–18.31)	9.82 (3.59–22.48)	0.415
Lymphocyte count (G/l)	1.64 (0.62–6.70)	1.4 (0.22–5.21)	0.079
Urea (mmol/l)	6.1 (2.6–107.0)	5.8 (2.4–28.1)	0.782
Creatinine (μmol/l)	61 (22–110)	58.0 (4.4–141.0)	0.974
Protein (g/l)	57.9 ± 6.5	56.2 ± 8.9	0.320
Albumin (g/l)	39.9 (31.2–44.4)	36.6 (20.1–53.1)	0.079
Alkaline phosphatase (U/l)	39 (3–268)	54 (10–1,008)	**0.024**
Alanine-Aminotransferase (U/l)	55 (12–457)	65 (19–852)	0.528
Glucose (mmol/l)	6.0 (4.4–7.5)	6.0 (2.5–12.0)	0.376
SNAP cPL (normal/abnormal)	29/7	43/22	0.169
Specific cPL (μmol/l)	657 ± 590	964 ± 668	0.290

Normally distributed values are presented as mean ± standard deviation, non-normally distributed values are presented as median and range. *p*-values in bold are significant.

cPL, canine pancreatic lipase.

#### 3.2.4. Ultrasound

The different gastric wall thickness measures between the UN (0.33 cm ± 0.11 cm; *P* = 0.034) and the COM (0.37 ± 0.09 cm) groups had a significance; however, the total numbers barely show any measurable difference. The duodenal and colonic wall thicknesses did not differ between the groups (*P* = 0.211 and *P* = 0.968, respectively; Table 6).

**Table 6:** Comparison of ultrasonographic intestinal wall thickness in 34 dogs with uncomplicated vomiting and 65 dogs with complicated vomiting.

**Parameter**	**Uncomplicated**	**Complicated**	***p*-value**
Gastric wall (cm; mean ± SD)	0.33 ± 0.11	0.37 ± 0.09	**0.034**
Duodenal wall (cm; median, range)	0.41 (0.21–0.65)	0.39 (0.23–0.68)	0.211
Colonic wall (cm; median, range)	0.20 (0.-09–0.34)	0.20 (0.09–0.53)	0.968

The bold value is statistically significant.

#### 3.2.5. SNAP cPL

SNAP cPL was abnormal in 29/99 (29%) dogs (seven dogs from UN and 22 dogs from COM group; *P* = 0.169). Mean spec cPL values in dogs with abnormal SNAP cPL in the COM group (964 ± 668 μg/l) were not significantly different from those in the UN group (657 ± 590 μg/l; *p* = 0.290; Table 5).

## 4. Discussion

The choice of diagnostic tests for patients with vomiting remains empirical. Few studies have investigated the utility of diagnostic tests for dogs with vomiting. One study evaluated the utility of abdominal ultrasonography in vomiting dogs. However, thereby exclusively chronically vomiting patients were included (3). Another study had a retrospective design without a standardized diagnostic protocol (2). In contrast, this study had a prospective design and evaluated the diagnostic utility of different tests using a standardized diagnostic approach.

In the present study, a definite diagnosis was achieved by initial blood tests (bloodgas analysis, complete blood count, serum biochemistry profile) in only one dog, which is contrary to the results of a previous study in which a diagnosis by blood tests was possible in 13% of the cases (2). The present study was conducted in an emergency setting. Therefore, the utility of the initial blood tests, which can usually be performed in-house, was assessed. Further examinations, such as endocrine testing, diagnostics for infectious diseases and parameters for clinical chemistry analysis, such as bile acids or ammonia, were not part of the initial blood tests. It should be noted that in the aforementioned study, this kind of laboratory testing was also included in the assessment (2), which might explain the divergence.

A significant correlation between the mentation and the diagnostic value of blood examinations was found, suggesting that dogs with a reduced mentation tend to have more significant deviations in blood results. For example, loss of chloride, sodium, potassium, and bicarbonate due to profuse vomiting can cause electrolyte and acid-base imbalances which might impair mentation (4, 5).

Attending clinical signs showed a significant impact on the utility of blood tests. Hemorrhagic diarrhea and other clinical signs (PU/PD, dyspnea, tremor, pruritus) were associated with a better diagnostic utility compared to non-hemorrhagic diarrhea or absence of additional clinical signs. At total of 15/31 dogs (48%) with hemorrhagic diarrhea was diagnosed with AHDS, which is often accompanied by marked hemoconcentration in combination with normal or reduced protein and albumin levels (6, 7). Blood examinations can deliver important information in these cases.

There was a significant correlation between the patients' mentation and the utility of abdominal radiographs. In case of severely impaired mentation, or acute signs in association with abdominal distention, efforts should be made to rapidly exclude life-threatening conditions, such as foreign bodies, gastric dilatation and volvulus or intestinal volvulus. Therefore, abdominal radiography is generally recommended unstable patients with vomiting (8). In the present study, radiographs were mainly redundant (87/99) for reaching a diagnosis; however, they were often essential to rule out gastrointestinal obstructions.

In 4/99 (4%) cases, radiographs were misleading. In three of these patients (3/4; 75%), gastrointestinal obstruction was suspected through radiographs; however, it could not be confirmed by repeated radiography, sonography, or clinical course of disease. In one case, radiographs were interpreted as nonobstructive, although there was an obstructive foreign body. Numerous studies have evaluated the utility of radiographs in the diagnosis of gastrointestinal obstruction (9–12). The sensitivity of plain radiography for the diagnosis of small intestinal obstruction varied between 76.8 and 87%, while specificity varied between 82.2 and 90.8% (9). In the present study, radiography was diagnostic in 2/3 (67%) cases of foreign-body ileus, as well as in one case of ileal coprostasis.

Urinalysis was decisive in diagnosis of only one case and provided important information in 7/99 cases (5.1%, score ≤ 3). In a previous retrospective study, urinalysis enabled or assisted diagnosis in 12.2% of cases (2). The less number of dogs with metabolic or endocrine disorders (e.g., kidney or liver failure, diabetes mellitus and diabetic ketoacidosis) in the present study might have contributed to these divergent findings.

Sonography obtained the diagnosis of 3/99 patients (3%; one foreign body ileus, two pyometra), while it was not helpful for diagnosis (score = 4) in 60/99 (61%) cases. These results are similar to the results of two other studies, in which diagnosis was obtained *via* sonography in 4% and 9% cases, and where the same diagnosis would have been reached without sonography in 68.5% and 53% of the dogs, respectively (2, 3).

A previous study investigated the utility of abdominal ultrasonography in dogs with chronic vomiting. Significant associations between the diagnostic utility and the patients' age, the number of vomiting episodes per week, the duration of vomiting and presence of weight loss were detected (3). In the present study, the percentage of weight loss was not included into the analysis, as very few dogs had any weight loss. Yet, there was also a significant correlation between duration of vomiting and utility of abdominal sonography. Additionally, other than in the before mentioned study, there was a significant association between utility of ultrasound and the patients' appetite.

The most common sonographic signs of gastrointestinal inflammation are mild and diffuse thickening of the gastrointestinal walls, mostly without loss of layering, and mild enlargement of regional lymph nodes (13–15). These findings were also present in some cases with no definite diagnoses (14/39, 35%). It can be assumed that acute gastroenteritis caused vomiting in many of these cases. However, without endoscopy, no definite proof of this suspicion exists and the diagnosis of these cases remains unclear.

SNAP cPL was abnormal in 29/99 (29%) cases. This test is very sensitive (91.5–94.1%) but not very specific (71.1–77.5%) for pancreatitis (16). As no single assay has a specificity high enough to diagnose pancreatitis on its own, recent study suggested the combination of clinical presentation, blood results and abdominal sonography to establish a definite diagnosis of pancreatitis (17). Therefore, in this study the tentative diagnosis of pancreatitis was confirmed when dogs showed elevated spec cPL levels and ultrasonographic evidence of pancreatitis. A negative SNAP cPL occurred in 70/99 dogs (71%) and is highly predictive of the absence of pancreatitis, but false-negative results may occasionally occur (18). As spec cPL was only measured in dogs with a positive SNAP cPL, the number of pancreatitis cases as an underlying cause of vomiting could have been underestimated. On the other hand, three dogs with ultrasonographic pancreatic changes and elevated spec cPL levels made an uncomplicated recovery within a few days. This could retrospectively challenge the initial diagnosis and raise the question if pancreatitis as an underlying cause of emesis could also have been overestimated.

Several criteria differed significantly between the UN and COM groups. Concurrent clinical signs, other than diarrhea, were more frequent in the COM group. Vomiting is often accompanied by diarrhea in cases of enteritis (15). A survey of 772 dogs showed that over a 2 week period, vomiting was present in 18.9% and diarrhea in 14.9% of dogs. Most of these episodes were self-limiting (1). When diarrhea was hemorrhagic, dogs were mostly in group COM (26/31; 84%). Approximately half of the dogs with hemorrhagic diarrhea (15/31; 48%), were diagnosed with AHDS and all of them had complicated vomiting. The etiology of this syndrome remains unknown and it is a diagnose of exclusion (6, 19, 20). Here, the diagnosis AHDS was made based on the typical presentation and clinical course of the dogs. All dogs diagnosed with AHDS had negative fecal examinations and a sufficient vaccination status. Thus, parasitic or parvoviral infections were unlikely. However, other underlying problems, e.g., viral or bacterial infections not tested for in this study, cannot be fully excluded.

Dogs presenting with impaired mentation were more likely to be in the COM group, and abdominal tension and pain score were higher in the COM group. Additionally, most of the dogs with abnormal body-temperature and all dogs with shock were assigned to the COM group. The decision to hospitalize a patient was made by the attending clinicians and was based on their subjective clinical assessment of the dog. It is safe to assume that most clinicians would recommend inpatient admission for dogs in shock, with impaired mentation, severe abdominal pain, or strongly deviant rectal temperature rather than sending them home with supportive treatment. Possibly, some hospitalized patients would also have recovered at home, so these findings could be biased.

### 4.1. Limitations

Dogs were classified as having UN if they recovered within a few days of presentation with symptomatic treatment only. As follow-up was performed only once, it cannot be excluded that some dogs showed relapse after the follow-up call.

This study was conducted in an internal medicine emergency department. Accordingly, no dogs that obviously required surgical intervention were presented. Dogs with witnessed intake of foreign bodies, with copremesis, or large breed dogs with massive, fast-growing abdominal distention, suspicious for gastric dilatation volvulus, are commonly presented directly to a clinic with surgical facilities. Therefore, such patients were not included in this study. This might have reduced the number of obstructive diseases, such as intestinal foreign bodies, intussusception, volvulus, or other conditions requiring surgical intervention. If the same study is performed in a surgical or mixed clinic, other results could be achieved. On the other hand, another study investigating causes of vomiting in dogs found gastrointestinal foreign bodies in 10/213 dogs (4.7%) (2), which is comparable to the present study, where obstructive foreign bodies (3/99; 3%) and adhesive ileus (1/99; 1%) were also uncommon diagnoses.

The utility of blood examinations might have been underestimated because there were no cases of severe azotemia or parvovirosis in the study population. As these conditions show typical deviations in different blood parameters and can lead to vomiting, blood tests would have been rated very useful in these cases. Although parvovirosis is rarely seen in Germany (21), where the study was performed, this infectious disorder might be common in other areas and thus specific tests for diagnosis and identification of complicating factors of parvovirosis (e.g., neutropenia) are indicated in every dog under 6 months of age as well as in every unvaccinated or inadequately vaccinated dog with acute gastrointestinal signs.

The quality of abdominal sonography and the accuracy of abdominal radiograph evaluation are strongly dependent on the experience and skills of the examiner. In the present study, non-board-certified emergency clinicians performed and evaluated the abdominal imaging. Although these veterinarians had comparable qualifications and training, their work experience and skills differed. This might have led to bias in the assessment of diagnostic utility, or even misdiagnosis. Nevertheless, daily clinical routine should be represented by the study design. In emergency services, particularly during night shifts, personnel are reduced and specialists are mostly not available.

The median utility was similar in the different diagnostic tests and every test was redundant in a high number of cases. This might follow from the high number of patients without a definite diagnosis. However, evaluating the diagnostic utility of a test does not give any information about its importance to exclude severe underlying causes. Consequently, a test that was rated low in diagnostic utility, can still be decisive to rule out possible life-threatening conditions like severe azotemia, anemia, intestinal foreign bodies and others.

As previously mentioned, *p*-values were not adjusted for multiple comparison between UN and COM because of the explorative nature of these tests. Therefore, the results must be interpreted with caution, and further investigations are needed to confirm their integrity.

### 4.2. Conclusion

The median utility score was similar for the evaluated diagnostic tests.

Factors that correlated with greater utility were impaired mentation, and attending clinical signs other than diarrhea for the initial blood examinations. Impaired appetite and a longer duration of vomiting were identified as correlating factors for abdominal sonography.

Patients with additional clinical signs other than diarrhea and dogs presenting with impaired mentation, shock, or severe abdominal pain were more likely to have a complicated disease course.

Based on the findings of this study, it seems reasonable to include ultrasonography into the diagnostic plan for dogs with impaired appetite and a longer duration of vomiting.

Referring to the large number of dogs where diagnostic tests were redundant, it seems legitimate to not standardly do further tests in clinically unremarkable dogs, particularly if they do not show attending clinical signs. However, to exclude severe underlying conditions and to meet the owners' wishes and concerns, basis testing are still often required.

Owing to the broad spectrum of underlying causes of emesis in dogs, reliable recommendations cannot be made for all vomiting patients, and further workup needs to be undertaken.

## Data availability statement

The original contributions presented in the study are included in the article/Supplementary material, further inquiries can be directed to the corresponding author.

## Ethics statement

The animal study was reviewed and approved by Ethics Committee of the Center of Clinical Veterinary Medicine. Written informed consent was obtained from the owners for the participation of their animals in this study.

## Author contributions

RD, BH, and SU: conceptualization, investigation, and methodology. BH: data curation and writing—original draft. BH and RD: formal analysis. RD: supervision. MW, SU, and RD: writing—review and editing. All authors contributed to the article and approved the submitted version.
