# Supplementary material for: Utility of diagnostic tests in vomiting dogs presented to an internal medicine emergency service
Source: Front Vet Sci. 2023 Feb 2;10:1063080. doi: 10.3389/fvets.2023.1063080 (PMC9933778; doi:10.3389/fvets.2023.1063080)

Figure 2. Correlation between appetite and utility score of abdominal sonography in 99 dogs with vomiting.


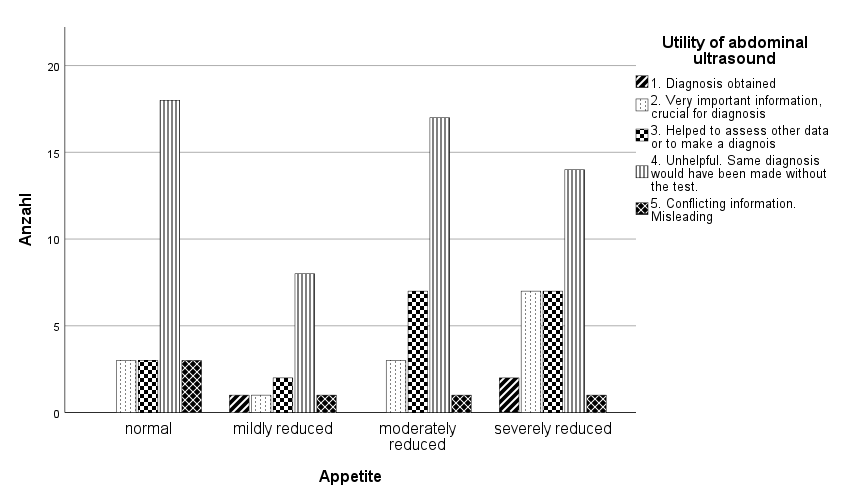


Figure 3. Comparison of mentation in 34 dogs with uncomplicated (UN) vomiting and 65 dogs with complicated vomiting (COM)


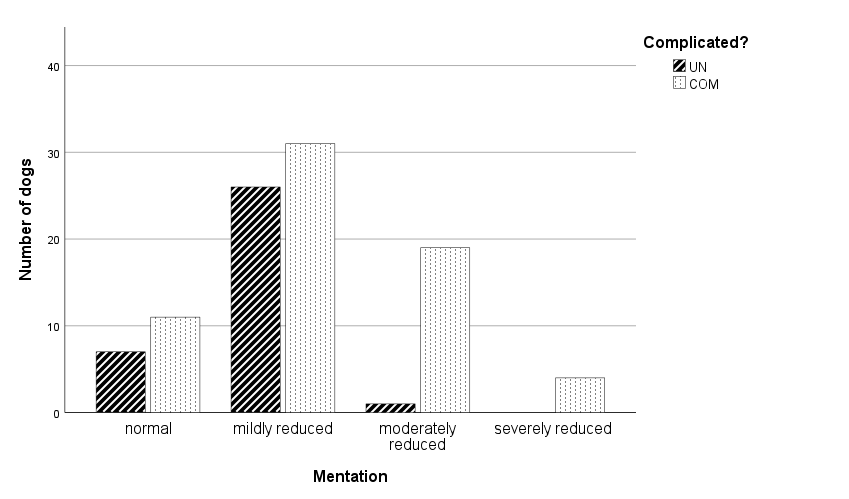


Figure 4. Comparison of pain scores in 34 dogs with uncomplicated (UN) vomiting and 65 dogs with complicated vomiting (COM)


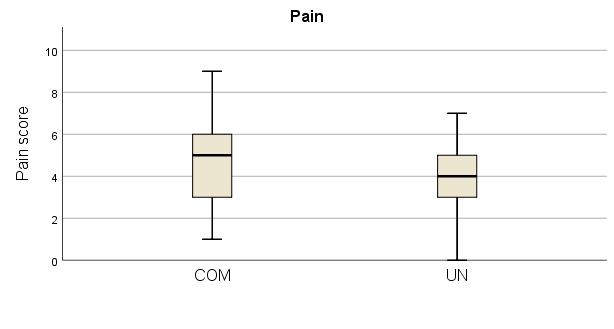


Figure 5. Comparison of heart rates in 34 dogs with uncomplicated (UN) vomiting and 65 dogs with complicated vomiting (COM)


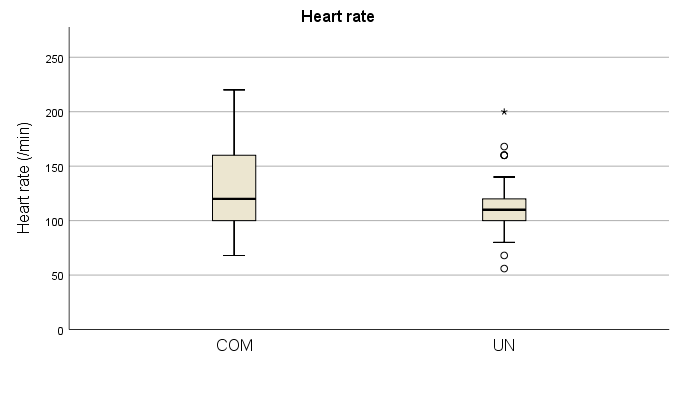

Supplement: Supplementary file 1 [file Data_Sheet_1.docx]
